# Phenytoin Is Promoting the Differentiation of Dental Pulp Stem Cells into the Direction of Odontogenesis/Osteogenesis by Activating BMP4/Smad Pathway

**DOI:** 10.1155/2022/7286645

**Published:** 2022-04-21

**Authors:** Wei Shang, Shijiang Xiong

**Affiliations:** Department of VIP Center, School and Hospital of Stomatology, Shandong University & Shandong Provincial Key Laboratory of Oral Tissue Regeneration & Shandong Engineering Laboratory for Dental Materials and Oral Tissue Regeneration, Jinan, 250012 Shandong, China

## Abstract

**Background:**

The purpose of this study was the evaluation of the potential and mechanism of phenytoin to promote differentiation of human dental pulp stem cells (hDPSC) into odontoblasts/osteoblasts.

**Methods:**

Fourth-generation human hDPSC originating from healthy pulp of third molars was cultured in control as well as phenytoin-containing media (PHT) for 14 days. qPCR was applied to detect the expression of DSPP, DMP1, and ALP genes. Western blot analysis was used to confirm the findings. One-way analysis of variance (ANOVA) was used for statistical analysis (*p* < 0.05). Information about phenytoin was assessed from PubChem database, while targets of phenytoin were assessed from six databases. Drug targets were extracted based on the differentially expressed genes (‖logFC‖ ≥ 1, *p* < 0.05) in the experimental group (50 mg/L PHT, 14 days). GO BP and KEGG pathway enrichment analysis on the obtained drug targets was performed and the target protein functional network diagram was constructed.

**Results:**

A concentration below 200 mg/L PHT had no obvious toxicity to hDPSC. The expression of DSPP, DMP1, and ALP genes in the 50 mg/L PHT concentration group increased significantly. The WB experiment showed that the protein content of BMP4, Smad1/5/9, and p-Smad1/5 was significantly increased in 50 mg/L PHT in comparison with the NC group (the group without treatment of PHT) at 14 days.

**Conclusion:**

Phenytoin has the ability of promoting the differentiation of hDPSC into odontoblasts and osteoblasts. BMP4/Smad pathway, inducing odontogenic/osteogenic differentiation of hDPSC, appears a main process in this context.

## 1. Introduction

Human dental pulp stem cells (hDPSC) are characterized to be highly clonogenic, having both multidifferentiation as well as neurovascular properties; thereby, hDPSC are the main actors in pulp homeostasis and regeneration [1]. Considering their functional abilities, hDPSC are examined to be a potential stem cell-based therapy for regeneration of the dental pulp and for peripheral nerve injury [2]. Generally, hDPSC are of neural crest origin; therefore, these cells have a large capacity to differentiate in a variety of tissues and a high plasticity, predisposing them for respective regenerative therapy approaches in- and outside of the oral cavity [3]. The understanding of hDPSC is difficult and complex. On the one hand, the pulpal inflammatory microenvironment interrelates with their proliferation and differentiation abilities [4]. On the other hand, the cellular senescence of hDPSC is crucial for their understanding and clinical implications, making increased knowledge on the molecular processes related to senescence in hDPSC needed [5].

A topic of particular interest is the differentiation of hDPSC into the direction of odontogenesis/osteogenesis. hDPSC have the potential to differentiate in both, bone and dental tissues, what has been demonstrated more than twenty years ago [6]. It is known that hDPSC can differentiate in odontoblast-like cells after induction of mineralization, what was confirmed in vitro [7, 8]. Thereby, different approaches were applied to promote the odontogenic differentiation of hDPSC. Endothelin-1, which is secreted by endothelial cells, was found to induce odontoblastic differentiation of hDPSC [9]. Similarly, nitric oxide was found to promote odontogenic differentiation in hDPSC [10]. Additionally, different materials used in clinical context, i.e., mineral trioxide aggregate (MTA), Biodentine and Emdogain were found to affect hDPSC differentiation in vitro [11].

Another approach for induction of differentiation is the use of phenytoin, an antiseizure drug acting at the voltage-gated sodium channel; phenytoin can induce several obscure mechanisms in different diseases, including breast cancer or optic neuritis [12]. Clinically, phenytoin has been repeatedly described related to its induction of gingival overgrowth [13]. However, it has also been examined with regard to osteogenic potential of hDPSC. A study by Asgharian-Rezaee et al. found phenytoin to show an osteogenic activity, inducing osteogenic differentiation of hDPSC [14]. Accordingly, phenytoin could also be a promising inductor for the differentiation of hDPSC into the direction of odontogenesis/osteogenesis. Up until now, it has not been reported whether phenytoin has the potential to induce the differentiation of hDPSC into odontoblasts/osteoblasts. Therefore, this current study aimed in the evaluation of the potential and mechanisms of phenytoin to promote the differentiation hDPSC into odontoblasts/osteoblasts by a two-step approach. Thereby, cell culture experiments were combined with a bioinformatics analysis to identify drug targets and related pathways.

## 2. Materials and Methods

### 2.1. Sample Collection

The pulp samples of healthy human third molars were obtained from three systemically healthy subjects (18-25 years of age), from the dentistry clinic of Heping Hospital Affiliated of Changzhi Medical College, Changzhi, China. The protocol was approved by the ethics committee, Changzhi Medical College (no: RT2021028), and the study was performed in accordance with the ethical standards of the Declaration of Helsinki. All donors signed informed consent.

### 2.2. Cells and Preparation

Caries-free third molars from these healthy patients were collected immediately following the extraction and were used to isolate hDPSCs. Dental pulp was isolated under sterile conditions and rinsed with PBS, after which they were minced using a ophthalmological scissors, and pulp aliquots were transferred into 6-well plates containing general medium (GM) composed of *α*-MEM containing 10% FBS and 1% penicillin-streptomycin (Gibco, USA). Cells were cultured in a 37°C with 5% CO_2_ incubator, with media being changed every other day until cells reached confluence, at which time cells were passaged. Cells were used for experimentation following 3-5 passages.

### 2.3. Alizarin Red and Oil Red O Staining

For osteogenic and adipogenic induction, third-passage hDPSCs were cultured in osteogenic and adipogenic differentiation media (Cyagen, USA), respectively. Next, cells were washed twice with phosphate-buffered saline (PBS) and fixed with 4% paraformaldehyde for 1 h. After fixation, cells were stained with Alizarin Red or Oil Red O for 30 min. Cells were observed and imaged by an inverted phase-contrast microscope (Olympus, Japan).

### 2.4. Colony Formation Assay

hDPSCs were seeded into 6-well dishes at a density of 500 cells/well. For the next 14 days, culture media was replaced every 3 ~ 4 days until visible colonies had developed. The colonies were washed with 1 × PBS, fixed with 4% paraformaldehyde and stained with 0.5% crystal violet. Colonies containing more than 50 cells were counted under a light microscope.

### 2.5. Flow Cytometry

After isolation and culture, the third-generation stem cells were collected and analyzed by flow cytometry using BD FACSMelody (BD Biosciences, San Diego, USA). Flowjo_v10 software was used to analyze the data. Thereby, FITC anti-CD90 (cat 328107), PE anti-CD105 (cat 800503), APC anti-CD73 (cat 344005), PerCP/Cy5.5 anti-CD34 (cat 343521), and APC/FireTM750 anti-CD45 (cat 368517) antibody (BioLegend, USA) stains were assessed for third-generation hDPSC.

### 2.6. Cell Proliferation Analysis

Cell viability was determined using Cell Counting Kit-8 (CCK-8, Dojindo, Japan). Briefly, hDPSCs were plated onto 96-well plates with 3000 cells/well. Cell proliferation capacity was evaluated at 1, 2, 3, 4, and 5 days by detecting the absorbance at 450 nm using a plate reader.

### 2.7. Alkaline Phosphatase Staining

After differentiation, cultured cells were stained by ALP staining kit (Solarbio, China) as per manufacturer's protocol. Briefly, the media were carefully removed from the cultured cell wells. Cells were fixed with 4% paraformaldehyde for 30 min at room temperature. Then, 500 *μ*l of wash buffer was gently added and carefully removed using a pipette. 250 *μ*l of ALP staining reagent solution was carefully added to completely cover the cells in each well of a 24-well plate. Cells were incubated for 30 min at 37°C before washed gently with 500 *μ*l of wash buffer for 3 times. 300 *μ*l of wash buffer was added, and stained cells were imaged using a light microscope.

### 2.8. Quantitative Polymerase Chain Reaction

In quantitative PCR (q-PCR) analysis, three experiments were implemented. First, total RNA was obtained using TRIzol Reagent (Introgen, CA, USA) and then reversely transcribed into cDNA using RevertAid First Strand cDNA Synthesis Kit (Thermo Fisher, IL, USA). qPCR was performed using SYBR Green PCR Master Mix (Applied Biosystems, Foster City, CA, USA) based on the 2^-△△Ct^ method. GAPDH was internal control.

### 2.9. Western Blot Analysis

Cell lysates prepared with RIPA lysis buffer (Thermo Fisher Scientific) were incubated on ice for 30 min, subjected to SDS-PAGE assay, and then transferred to nitrocellulose membranes. Western blot analysis was conducted as described previously. Briefly, cell lysates were separated on 10% SDS-PAGE gels, transferred to 0.45 *μ*m pore-size PVDF membranes, blocked with 5% BSA, and then incubated with a panel of antibodies, including those against hDSPP, hDMP1, GAPDH, hBMP4, Smad1/5/9, and p-smad1/5. The membranes were then incubated with HRP-conjugated secondary antibodies (1 : 5000) at room temperature for 1 hour then visualized using an ECL staining kit (Applygen, China). Anti-glyceraldehyde-3-phosphate dehydrogenase (GAPDH) antibody acted as a loading control.

### 2.10. RNA-Sequencing

Regarding the first step of RNA quantification and qualification, RNA integrity was assessed using the RNA Nano 6000 Assay Kit of the Bioanalyzer 2100 system (Agilent Technologies, CA, USA). For the second step of library preparation for transcriptome sequencing, total RNA was used as input material for the RNA sample preparations. Briefly, mRNA was purified from total RNA using poly-T oligo-attached magnetic beads. Fragmentation was carried out using divalent cations under elevated temperature in first-strand synthesis reaction buffer (5X). First -strand cDNA was synthesized using random hexamer primer and M-MuLV Reverse Transcriptase (RNase H-). Second strand cDNA synthesis was subsequently performed using DNA Polymerase I and RNase H. Remaining overhangs were converted into blunt ends via exonuclease/polymerase activities. After adenylation of 3′ ends of DNA fragments, adaptor with hairpin loop structure was ligated to prepare for hybridization. In order to select cDNA fragments of preferentially 370〜420 bp in length, the library fragments were purified with AMPure XP system (Beckman Coulter, Beverly, USA). Then, PCR was performed with Phusion High-Fidelity DNA polymerase, Universal PCR primers, and Index (X) Primer. At last, PCR products were purified (AMPure XP system), and library quality was assessed on the Agilent Bioanalyzer 2100 system. Afterward, clustering and sequencing were carried out. The clustering of the index-coded samples was performed on a cBot Cluster Generation System using TruSeq PE Cluster Kit v3-cBot-HS (Illumia) according to the manufacturer^ instructions. After cluster generation, the library preparations were sequenced on an Illumina Novaseq platform and 150 bp paired-end reads were generated.

### 2.11. Differential Expression Analysis Based on the RNA-Seq Results

Differential expression analysis of two conditions/groups (two biological replicates per condition) was performed using the DESeq2 R package (1.20.0). DESeq2 provides statistical routines fbr determining differential expression in digital gene expression data using a model based on the negative binomial distribution. The resulting *p* values were adjusted using the Benjamini and Hochberg's approach for controlling the false discovery rate. Genes with an adjusted *p* value < 0.05 found by DESeq2 were assigned as differentially expressed.

### 2.12. GO and KEGG Enrichment Analysis of Differentially Expressed Genes (DEGs)

Gene Ontology (GO) enrichment analysis of differentially expressed genes was implemented by the clusterProfiler R package, in which gene length bias was corrected. GO terms with corrected *p* value less than 0.05 were considered significantly enriched by DEGs. KEGG is a database resource fbr understanding high-level functions and utilities of the biological system, such as the cell, the organism, and the ecosystem, from molecular-level information, especially large-scale molecular datasets generated by genome sequencing and other high-throughput experimental technologies (http://www.genome.jp/kegg/). We used clusterProfiler R package to test the statistical enrichment of differential expression genes in KEGG pathways. In addition, PPI analysis of DEGs was carried out based on the known and predicted Protein-Protein Interactions of the STRING database.

### 2.13. Phenytoin Drug Target Prediction

Information about phenytoin was assessed from PubChem database (Table 1). The targets of phenytoin were predicted based on six databases: SwissTargetPrediction, STITCH, Drugbank, TTD, PharmMapper, and CTD.

### 2.14. Screening of Drug Targets

In order to analyze the role of phenytoin sodium in hDPSC odontogenic/osteogenic differentiation, drug targets were extracted. This was based on the differentially expressed genes (‖logFC‖ ≥ 1, *p* < 0.05) in the experimental group (50 mg/L PHT, 14 days). The differentially expressed genes obtained from the gene expression profile and the phenytoin drug targets were crossed, and the obtained genes were the drug targets of phenytoin affecting hDPSC odontogenic/osteogenic differentiation.

### 2.15. The Functional Analysis of the Drug Target

The clusterProfiler of R program was used to perform GO BP and KEGG pathway enrichment analysis on the obtained drug targets. Enrichment functions with a *p* < 0.05 were significant. In addition, we used Cytoscape plug-ins ClueGO and CluePedia to analyze the drug targets of phenytoin influencing hDPSC odontogenic/osteogenic by GO biological process analysis and used Cytoscape as the target protein functional network diagram. In the case of using data without a hierarchical structure (KEGG, BioCarta), its level was specified as -1. The Kappa coefficient was used for consistency testing and to measure classification accuracy. The calculation of Kappa coefficient was based on a confusion matrix. In the ClueGO plug-in, the Kappa coefficient shows the relationship between GO terms based on overlapping genes. The higher the Kappa coefficient, the stronger the correlation between GO terms.

### 2.16. Phenytoin in the Drug-Target-Protein-Interaction Network

Based on BIOGRID (Biological General Repository for Interaction Datasets), HPRD (Human Protein Reference Database), DIP (Database of Interacting Proteins), MINT (Molecular INTeraction database), PINA (Protein Interaction Network Analysis), InnateDB (a knowledge resource for Innate immunity interactions & pathways), and Instruct (3D protein interactome networks with structural resolution), drug-target-protein-interaction relationship pairs were extracted. Cytoscape software was used to construct a PPI network.

### 2.17. Molecular Docking of Phenytoin and Dentin Differentiation Target Proteins

The software tools related to AutoDock were downloaded from http://mgltools.scripps.edu/downloads and http://vina.scripps.edu/download.html, respectively. The AutoDock molecular docking of the obtained drug target of phenytoin that affects hDPSC odontogenic/osteogenic differentiation and phenytoin was carried out. Afterward, the software Chem3D was applied to optimize the structure of the small molecule ligand. Then, the software AutoDockTool was used to convert the saved file to pdbqt format.

The data from UniProt regarding the protein receptors of the drug target of phenytoin affecting hDPSC odontogenic/osteogenic differentiation were downloaded. Then, the software pymol (https://pymol.org/2/) was used to delete the water molecules and small molecule ligands in the protein receptor. Moreover, the software AutoDockTool was applied to process the preserved protein receptors. The pdbqt format files of small molecule ligands and protein receptors were obtained, respectively. Furthermore, grid parameter GPF files were obtained. Using Vina and these three files, AutoDock molecular docking was performed.

### 2.18. Data Analysis

For analysis, GraphPad Prism (version 8.0; USA) was used. The data were expressed as the average of three independent experiments. One-way analysis of variance (ANOVA) was used to compare the average of the outcome variables of each categorical variable group. *p* < 0.05 was considered statistically significant.

## 3. Results

### 3.1. Study Design of the Current Research

**Figure 1:**
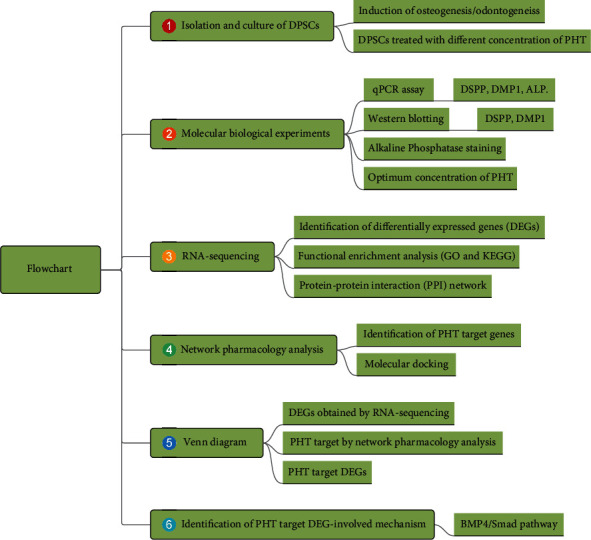
The flowchart of the current research.

As shown in Figure 1, the present research combined many techniques: molecular biology experiments, RNA-sequencing analysis, as well as network pharmacology and molecular docking. First, DPSCs were isolated from pulp tissue and further cultured. Second, a series of molecular biological experiments (e.g., qPCR assay, western blotting, and ALP staining) were carried out in order to identify the optimum concentration of PHT. Third, RNA-sequencing assay was performed to identify the differentially expressed genes (DEGs) when comparing the PHT-treated DPSC group and PHT-untreated DPSC group. Based on these DEGs, the functional enrichment analysis was performed, and protein-protein interaction (PPI) network was constructed. Fourth, network pharmacology analysis was performed to identify the PHT target genes. Fifth, a Venn diagram was constructed by overlapping DEGs obtained by RNA-seq and PHT target genes obtained by network pharmacology analysis, in order to identify the PHT target DEGs. Finally, BMP4 was selected among the PHT target DEGs to be investigated, and a specific BMP4/Smad signaling axis was identified to be involved in the PHT-induced osteogeneic/odontogenic differentiation.

### 3.2. Cell Culture Results

**Figure 2:**
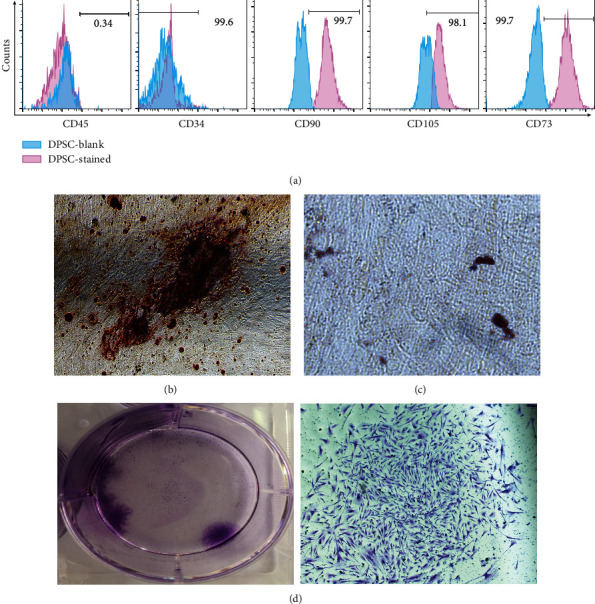
Stem cell identification results. (a) Flow cytometry was used to detect the molecular markers of the third generation of hDPSCs, and the positive expression rates of CD45 were 0.34%, the positive expression rates of CD34 were 0.4%, the positive expression rates of CD90 were 99.7%, the positive expression rates of CD105 were 98.1%, and the positive expression rates of CD73 were 99.7%. (b) Third-passage hDPSCs were stained with Alizarin Red 28 days after osteogenic induction, and the results showed that a large number of mineralized nodules were formed. (c) Third-passage hDPSCs were stained with Oil Red O 21 days after the induction of adipogenesis, and the results showed lipid droplets forming. (d) The colony formation experiment results show the cells were cultured for 14 days, and the clonogenesis rate was 5-15 cells/1000 cells (number of cells ≥ 50).

The flow cytometer showed that the cell surface markers used were negative for CD45/CD34 and positive for CD90, CD105, and CD73 (Figure 2(a)). The results showed that the detected cells were stem cells. 21 days after induction of adipogenesis, the hDPSC became adipocyte-like cells (Figure 2(c)); and the cells were stained with red lipid droplets indicating the multidifferentiation potential of hDPSC and to prove their dryness. 28 days after osteogenic induction, mineralized nodules were seen in the cells (Figure 2(b)). The colony formation experiment showed that cells had a strong ability to proliferate and form colonies, which indirectly proves the existence of stem cells (Figure 2(d)).

**Figure 3:**
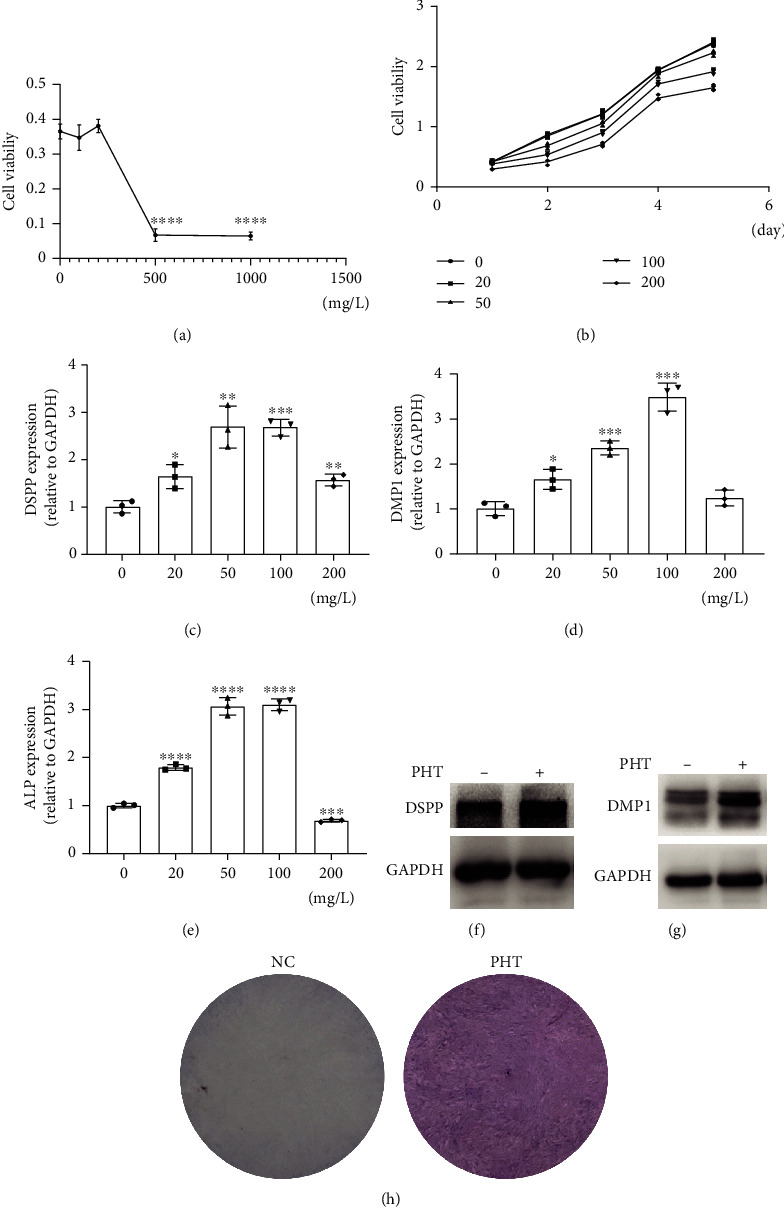
CCK-8 experiment results and the expression of odontogenic/osteogenic genes and proteins at different drug concentrations on 14 days. hDPSCs were cultured in general medium containing different concentrations of PHT for 14 days. Proteins and mRNAs were collected on day 14 after induction for Western blot and qRT-PCR. (a) CCK-8 cytotoxicity experiment showed the concentration below 200 mg/L PHT had no obvious toxicity to hDPSC. (b) CCK-8 cell proliferation experiment showed that the inhibitory effect of hDPSC proliferation increased with the higher PHT concentration. (c)–(e) show the expression of DSPP, DMP1, and ALP genes at different drug concentrations on 14 days, and related genes were elevated in the 50 mg/L and 100 mg/L PHT concentration. (f, g) Western Blot experiment showed the protein expression levels of DSPP and DMP1 were significantly upregulated in the 50 mg/L PHT concentration. (h) ALP staining showed an increase in the 50 mg/L PHT group on 14 days.

CCK-8 cytotoxicity experiment showed that the 500 mg/L PHT concentration group had obvious cytotoxicity at 6 h, and the concentration below 200 mg/L PHT had no obvious toxicity to hDPSC (Figure 3(a)). The CCK-8 cell proliferation experiment showed that the inhibitory effect of hDPSC proliferation increased with the higher PHT concentration (Figure 3(b)). The expression of DSPP, DMP1, and ALP genes at different drug concentrations (20 mg/L, 50 mg/L, 100 mg/L, and 200 mg/L) at 14 days, and related gene expression in the 50 mg/L and 100 mg/L PHT concentration groups increased significantly (Figures 3(c)–3(e)). Western blot showed that DSPP and DMP1 were significantly increased in comparison with NC group at 50 mg/L PHT at 14 days (Figures 3(f) and 3(g)). ALP staining was significantly darker in 50 mg/L PHT compared with NC group (Figure 3(h)).

### 3.3. hDPSC Differentially Expressed Genes for Odontoblastic/Osteoblastic Differentiation

**Figure 4:**
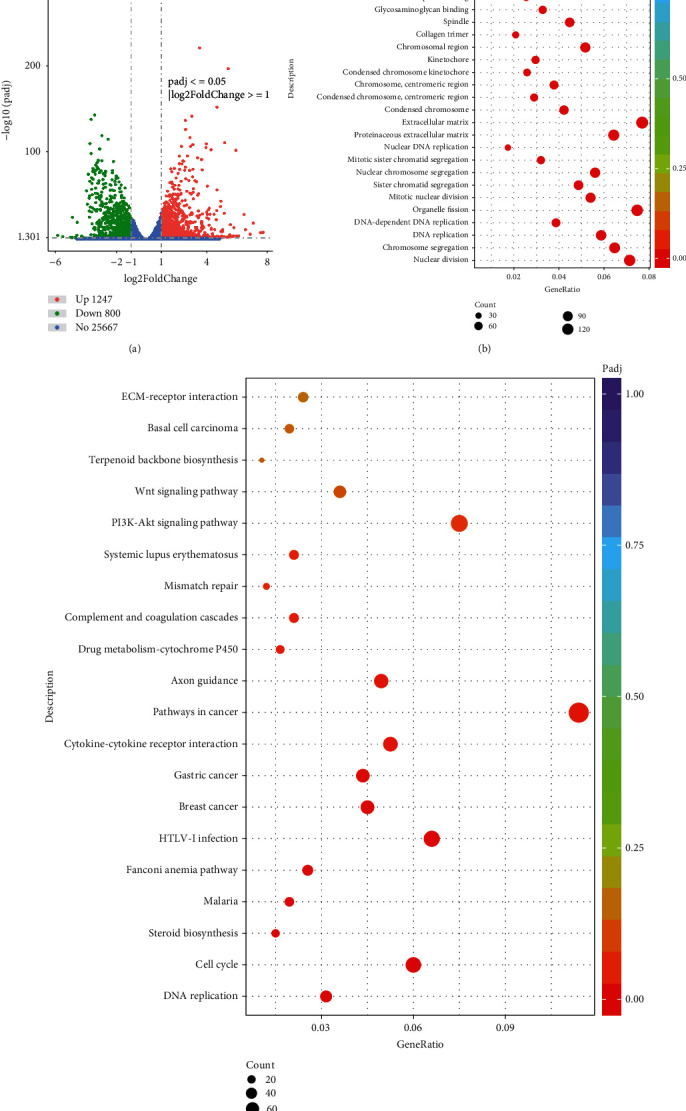
The DEGs dysregulated between PHT and NC group. (a) The volcano plot showing the expression pattern of DEGs in PHT and NC group. (b) The GO functional terms enriched by the DEGs expressed between PHT and NC group. (c) The KEGG signaling pathway enriched by the DEGs expressed between PHT and NC group.

Differentially expressed genes are displayed in a volcano map (Figure 4(a)). Genes differentially expressed in hDPSC odontogenic/osteogenic differentiation were mainly involved in biological processes such as nuclear division and chromosome segregation (Figure 4(b)). Among the biological pathways, the differentially expressed genes of hDPSC odontogenic/osteogenic differentiation mainly regulated biological pathways such as DNA replication and cell cycle (Figure 4(c)).

### 3.4. Phenytoin Drug Target Prediction

**Figure 5:**
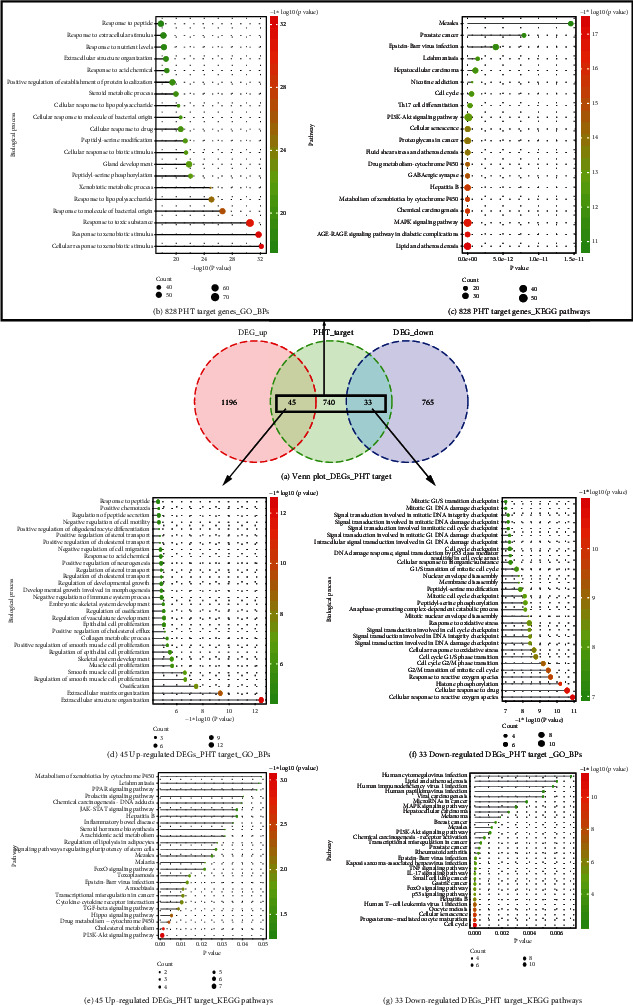
The overlapping genes between the DEGs and PHT target genes and these overlapping genes' enriched biological processes and KEGG pathways. (a) Venn diagram showing the overlapping genes between DEGs and PHT target genes. (b) The significantly enriched biological processes of the PHT target genes. (c) The significantly enriched signaling pathways of the PHT target genes. (d) The significantly enriched biological processes enriched by the upregulated PHT target DEGs. (e) The significantly enriched KEGG pathways enriched by the upregulated PHT target DEGs. (f) The significantly enriched biological processes enriched by the down-regulated PHT target DEGs. (g) The significantly enriched KEGG pathways enriched by the downregulated PHT target DEGs.

By querying the database and converting the protein name to the gene name, the phenytoin drug target data were finally obtained and shown in Table 2. Taking the union of the drug targets obtained from the abovementioned databases, and after deduplication, a total of 740 phenytoin-related drug targets were predicted. Clusterprofilter was used to perform functional analysis of drug targets, and the results showed that phenytoin is mainly involved in biological processes such as cellular response to xenobiotic stimulus, response to lipopolysaccharide and peptidyl-serine phosphorylation (Figure 5(b)). Thereby, phenytoin was involved in different biological pathways, mainly regulating lipid and atherosclerosis, MAPK signaling pathway, PI3K-Akt signaling pathway, and AGE-RAGE signaling pathway (Figure 5(c)).

### 3.5. The Drug Target of Phenytoin Regulating hDPSC Odontogenic/Osteogenic Differentiation

**Figure 6:**
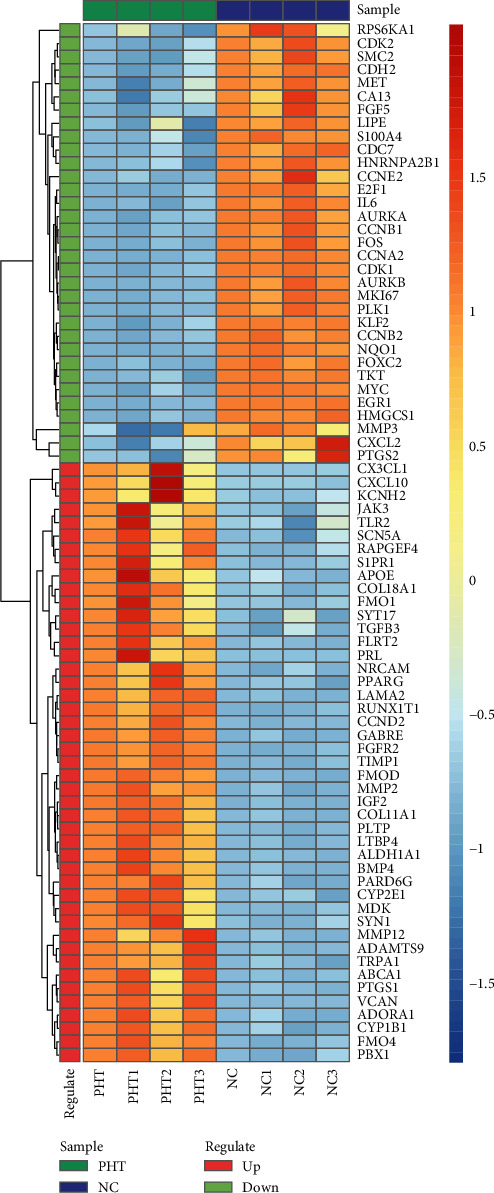
The heat map showing the expression pattern of 78 PHT target DEGs.

**Figure 7:**
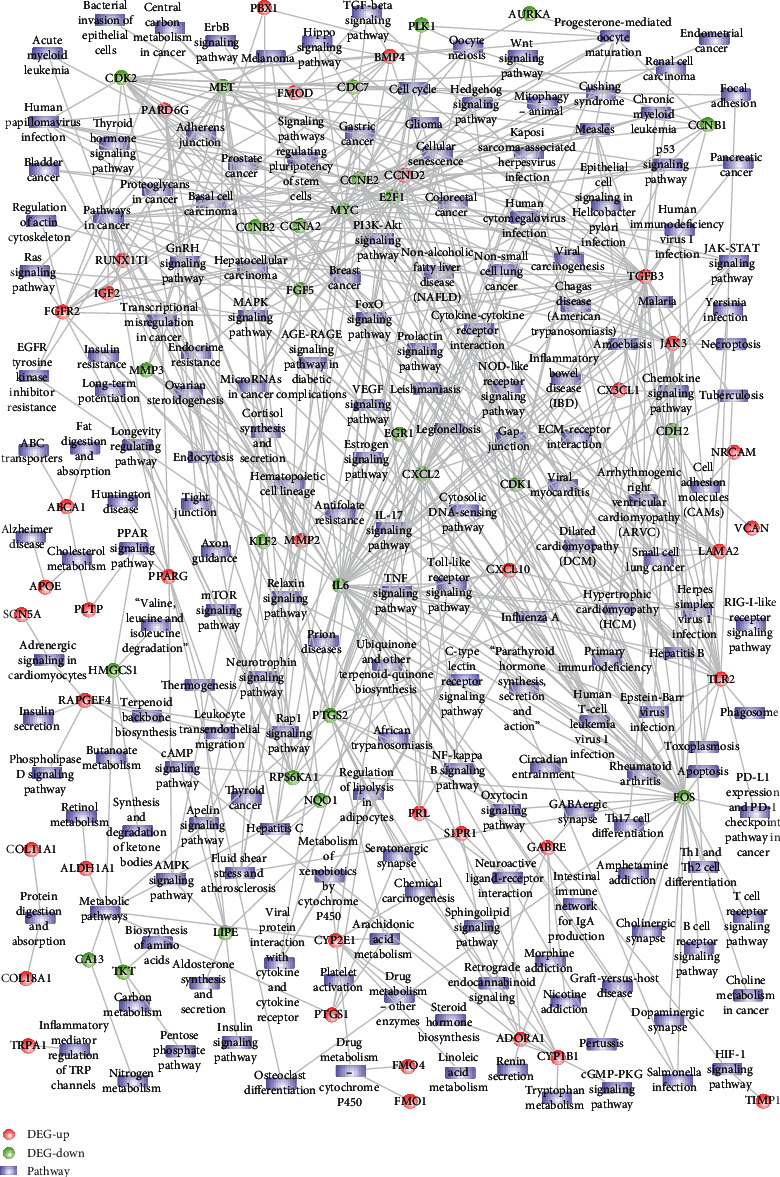
GO_BPs functional network analysis results showing the phenytoin affects the target of hDPSC ontogenetic/osteogenic differentiation.

The intersection of genes differentially expressed in hDPSC odontogenic/osteogenic differentiation and the drug targets of phenytoin were extracted. Thereby, 78 drug targets of phenytoin affecting dentin differentiation were obtained, of which 45 genes were upregulated and 33 genes were downregulated (Figure 5(a)). The 45 upregulated PHT target DEGs were mainly involved in biological processes such as extracellular structure organization and skeletal system development (Figure 5(d)) and regulated PI3K-AKt signaling pathway and TGF-beta signaling pathway (Figure 5(e)). The 33 downregulated PHT target DEGs were mainly involved in biological processes such as cellular response to reactive oxygen species (Figure 5(f)) and regulated pathways such as cell cycle and cellular senescence (Figure 5(g)). The heat map is showing the expression pattern of the overlapping genes between the DEGs (differentially expressed genes) and PHT target genes (Figure 6). In addition, GO biological processes particularly BPs related functional network analysis was performed on 78 PHT target DEGs (Figure 7).

### 3.6. The Drug Target Interaction Network of Phenytoin Regulating hDPSC Odontogenic/Osteogenic Differentiation

**Figure 8:**
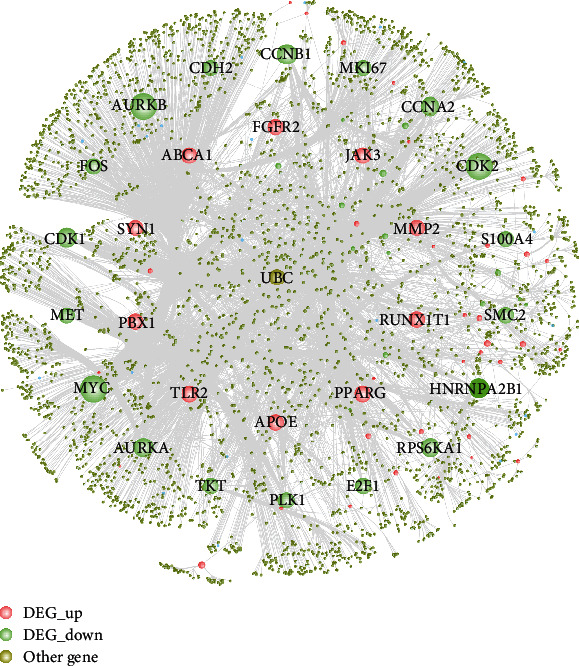
Phenytoin regulates the drug-target-interaction network of hDPSC ontogenesis/ontogenesis differentiation. The size of the nodes in the figure is sorted by the degree of network topology. The larger the node, the greater the degree of the gene.

A total of 5955 PPIs were obtained, which were drug targets of phenytoin to regulate hDPSC odontogenic/osteogenic differentiation from databases such as HPRD and used Cytoscape software to construct a PPI network, which included 3636 nodes and 5955 edges (Figure 8).

### 3.7. Molecular Docking of Related Odontogenic/Osteogenic Differentiation Target Proteins

**Figure 9:**
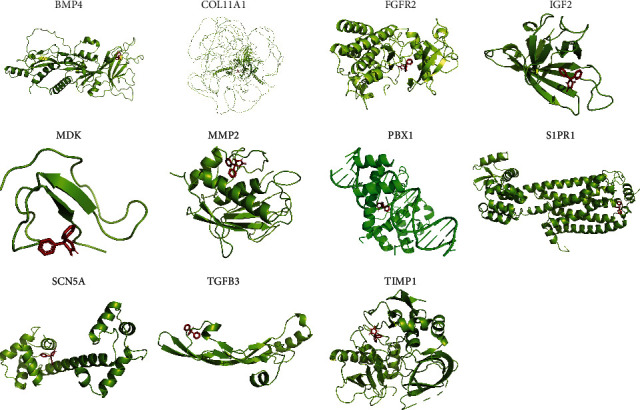
The three-dimensional molecular docking diagram of phenytoin with 11 proteins with information.

After inquiry, no protein structure information of VCAN was obtained. Therefore, the molecular docking of 11 informative proteins with phenytoin was carried out (Table 3 and Figure 9). These 11 proteins include BMP4, COL11A1, FGFR2, IGF2, MDK, MMP2, PBX1, S1PR1, SCN5A, TGFB3, and TIMP1.

### 3.8. Experimental Validation of a Significant Gene-BMP4 and Identification of BMP4-Related Pathway

**Figure 10:**
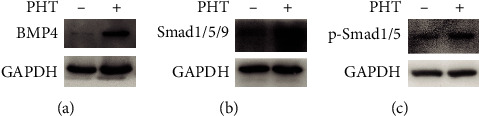
BMP4/Smad pathway verification results. (a) The protein level of BMP4 was increased in PHT (50 mg/L) group than in the NC group. (b) The protein level of Smad 1/5/9 was elevated in PHT (50 mg/L) group than in the NC group. (c) The protein level of p-Smad 1/5 was significantly upregulated in PHT (50 mg/L) group than in the NC group.

Western blot showed that the protein content of BMP4, Smad1/5/9, and p-Smad1/5 was significantly increased in comparison with the NC group at 50 mg/L PHT at 14 days, suggesting that PHT may upregulate and activate pathway-related factors promoting the differentiation of hDPSC towards odontogenic/osteogenic (Figure 10).  Figure 10(a) shows that the protein level of BMP4 was increased in PHT (50 mg/L) group than in the NC group.  Figure 10(b) shows that the protein level of Smad 1/5/9 was elevated in PHT (50 mg/L) group than in the NC group.  Figure 10(c) shows that the protein level of p-Smad 1/5 was significantly upregulated in PHT (50 mg/L) group than in the NC group.

## 4. Discussion

This current study showed that phenytoin at a suitable concentration can promote the differentiation of hDPSC into odontoblasts/osteoblasts. Compared with the control group, the related odontogenic/osteogenic indices of the 50 mg/L PHT group were significantly increased. The results of RNA sequencing showed that phenytoin had a significant effect on hDPSC. The intersection with the results of network pharmacology analysis revealed 45 upregulated genes and 33 downregulated genes. BMP4, which was highly related to tooth development, was selected for experiment. A one-step verification test found that the BMP4 protein was significantly increased compared with the control group, the related factors of the Smad pathway were significantly increased, and the p-Smad1/5 protein level was significantly increased compared with the control group. This suggests that phenytoin can upregulate and activate BMP4/Smad pathway to promote the differentiation of hDPSC into odontoblasts/osteoblasts.

This is the first study investigating the potential and mechanism of phenytoin to promote the differentiation of hDPSC into odontoblasts/osteoblasts. One previous study examined phenytoin regarding its osteogenic activity and showed that phenytoin achieved comparable findings as dexamethasone in inducing osteogenic differentiation of hDPSC [14]. Dexamethasone was described previously as a potent substrate inducing osteogenic differentiation of hDPSC [15]. A similar ability of phenytoin to induce osteogenic differentiation and therefore its potential in this context was also confirmed in the current study.

Odontogenic differentiation of hDPSC is a field of high clinical relevance, as it might be a key for understanding and application of regenerative approaches in dentistry. Thereby, inducing the differentiation of hDPSC into odontoblasts might be helpful in avoidance of progression of dental hard tissue diseases and thus preserving vitality of the dental pulp [16, 17]. Newly regenerated dental hard tissue has also the potential to repair dental damage [18]. Different studies examined the odontogenic differentiation of hDPSC and highlighted a variety of potentially related pathways, related genes, and interacting proteins [19–23]. This current study chooses phenytoin as focus of investigation to reveal a novel approach to induce this differentiation in hDPSC. Phenytoin has been shown to affect calcium homeostasis and/or bone metabolism before [14, 24]. However, the effect of phenytoin on the differentiation of hDPSC into odontoblasts has not been studied, yet; thereby, the current study confirmed this for the first time.

The BMP4/Smad pathway was revealed as potential key process in the differentiation of hDPSC induced by phenytoin. BMP4, i.e., bone morphogenic protein 4 is part of the BMP superfamily, including proteins with various functions in almost all aspects of bone, cartilage, and joint repair [25]. It is documented that BMP4 plays an important role during tooth morphogenesis and tooth formation; thereby, BMP4 signaling has the ability to suppress tooth developmental inhibitors and thus promoting tooth development [26]. Therefore, it is not conspicuous that BMP4 was also examined as an actor in odontogenic differentiation of hDPSC [27, 28]. The ability of phenytoin to affect expression of BMP4 has also been confirmed in animal model [29]. Moreover, literature suggests that BMP/Smad pathway is a mechanism of high relevance in osteogenic and ontogenetic differentiation of hDPSC [30]. Another study based on miRNA sequencing revealed TGF*β*1/Smad signaling pathway, where TGF*β*1 is in the same family as BMP, to promote odontogenic differentiation of hDPSC [21]. However, results reporting BMP4/Smad pathway in hDPSC differentiation are rare. Several other results support its role in osteogenic/odontogenic pathways. For example, overexpression of BMP4/Smad signaling pathway was related to chondroma of the skull [31]. Regardless, the finding of the current study that phenytoin affects osteogenic and ontogenetic differentiation of hDPSC due to BMP4/Smad pathway is novel and of potential clinical relevance, but needs further validation.

For the understanding of processes and pathways, PPI network analysis and evaluation of related pathways are of high interest in understanding hDPSC differentiation, as it helps to reveal the functions of identified proteins [32, 33]. Therefore, the current study applied comprehensive bioinformatics analysis. A previous differential expression and enrichment analysis found phosphoinositide-3-kinase-protein kinase (PI3K), TGF*β*, cell migration, cell differentiation, stem cell development, ossification, and skeletal development to be related to osteogenic and odontogenic differentiation in hDPSC [23]. Similar pathways were also found in deregulation of phenytoin-related hDPSC odontogenic/osteogenic differentiation drug targets in the current study. Altogether, recent results on pathways and processes related to osteogenic and odontogenic differentiation of hDPSC are various, heterogeneous, and complex; thereby, findings are mostly related to the substrate of induction [15, 19–23, 34, 35], making further comparisons with literature difficult. Based on the high clinical potential of hDPSC in regenerative therapy of dental diseases [16, 17], this emerging field of research is of high interest and the current study's findings provide several novel approaches regarding the use of phenytoin in this context.

This is the first study examining the suitability of phenytoin for differentiation of hDPSC into odontoblasts/osteoblasts. The study design was two-stepped, including cell culture and bioinformatics. The applied methods were comprehensive and valid, strengthening the current study's results and related conclusions. However, this examination was limited by its in vitro and bioinformatics design, whereby the clinical validation and potential practical usage of phenytoin need further validation. Especially the fact that bioinformatics results were on transcriptomic level must be considered as relevant factor. Further research is required to validate the novel results of odontogenic differentiation of hDPSC induced by phenytoin.

## 5. Conclusions

The data of this study show that phenytoin has the ability of promoting the differentiation of hDPSC into odontoblasts and osteoblasts. One major mechanism appears to be the upregulation and activation of the related factors of BMP4/Smad pathway, inducing odontogenic/osteogenic differentiation of hDPSC.

## Figures and Tables

**Table 1:** Information on phenytoin in the PubChem database.

	Phenytoin sodium	
PubChem CID	657302	1775
Molecular formula	C15H11N2NaO2	C15H12N2O2
Canonical SMILES	C1 = CC=C(C=C1)C2(C(=O)[N-]C(=O)N2)C3 = CC=CC=C3.[Na+]	C1 = CC=C(C=C1)C2(C(=O)NC(=O)N2)C3 = CC=CC=C3

**Table 2:** Phenytoin drug target prediction.

Database	Drug_targets
CTD	429
DrugBank	33
pharmMapper	269
STITCH	10
SwissTargetPrediction	120
TTD	1

**Table 3:** Docking results of the active components of phenytoin with odontogenic/osteogenic genes.

Protein	Ligand	Affinity (kcal/Mol)	Center_x	Center_y	Center_z	Size x	Size y	Size z
BMP4	CID1775	-7.2	21.346	15.236	-7.845	19	19	19
COL11A1		-7.4	16.664	-1.218	-17.799	32	25	19
FGFR2		-8.7	25.333	-5.704	1.311	19	19	19
IGF2		-6.4	-1.713	19.078	11.731	19	19	19
MDK		-6.1	-0.753	-2.399	14.09	19	19	19
MMP2		-6.8	1.572	-4.634	6.759	19	19	19
PBX1		-8.7	-0.72	24.956	7.494	34	25	29
S1PR1		-7.4	1.77	13.365	-10.656	26	19	27
SCN5A		-6.6	-1.108	2.056	22.412	19	19	19
TGFB3		-6.7	-21.547	20.42	23.061	19	19	19
TIMP1		-8.2	146.459	-7.049	4.149	19	19	19

## Data Availability

The data used to support the findings of this study are available from the corresponding author upon reasonable request.
